# EBM BLS: Beta-blockers Fail to Improve Cardiovascular Outcomes in Patients with Myocardial Infarction and Preserved Ejection fraction

**DOI:** 10.1007/s11606-026-10414-6

**Published:** 2026-05-14

**Authors:** William Beaty, Steven Allon

**Affiliations:** Division of General Internal Medicine, Vanderbilt University Medical Center, Nashville, TN USA

**Keywords:** myocardial infarction, coronary artery disease, beta blockers

**Source Article:** Yndigegn T, Lindahl B, Mars K, et al. Beta-Blockers after Myocardial Infarction and Preserved Ejection Fraction. *N Engl J Med*. 2024;390(15):1372-1381. doi:10.1056/NEJMoa2401479

## WHY THIS IS IMORTANT

- Despite substantial progress in medical and interventional therapy, coronary artery disease (CAD) remains the leading cause of death in the United States.^1^
- Early trials showed mortality benefit from beta-blockers after myocardial infarction (MI). However, these studies predated the advent of aspirin, percutaneous coronary intervention (PCI), statins, and P2Y12 inhibitors.
- Recent observational studies have not found an association between beta-blockers and mortality for patients with preserved ejection fraction (EF) post-MI.^2^
- The ABYSS trial found that discontinuing beta-blockers was associated with higher rates of adverse cardiac events; however, the number of deaths and MI was similar between groups and the difference in outcomes was driven by cardiac hospitalization.^3^

## INTERVENTION

**Figure 1:**
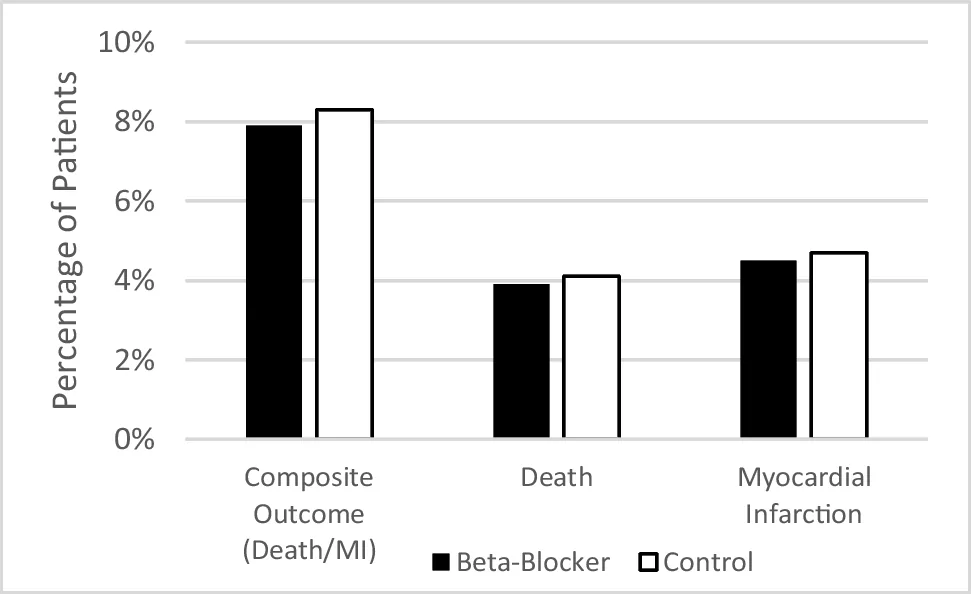
Death and myocardial infarction at follow-up.

- 5,020 participants were randomized in 1:1 ratio to beta-blockers (metoprolol or bisoprolol) or usual care.
- The primary endpoint was a composite of death from any cause or MI. Secondary outcomes were cardiovascular death, hospitalization for atrial fibrillation and heart failure.
- Safety endpoints included hospitalizations for stroke, bradycardia, atrioventricular block, hypotension, syncope, or pacemaker implantation (Fig. 1).

## RESULTS

- Median age was 65 years, 78% male, 35% presented with ST-elevation MI, and 95% were from Sweden.
- Most participants had single- or two-vessel disease (82.7%), and nearly all received PCI (95.8%).
- Participants were followed for a median of 3.5 years.
- Crossover occurred in 18.1% of participants in the beta-blocker arm and 14.3% in the usual care arm.
- The primary outcome of death or recurrent myocardial infarction occurred in 7.9% of beta-blocker patients and 8.3% of usual-care (HR 0.96; 95% CI 0.79–1.16).
- Secondary outcomes of cardiovascular death (1.5% vs 1.3%), hospitalizations for atrial fibrillation (1.1% vs 1.4%) and for heart failure (0.8% vs 0.9%) were similar.
- Safety endpoints were also similar including hospitalization for bradycardia, AV block, hypotension, or syncope (3.4% vs 3.2%), stroke (1.4% vs 1.8%), and hospitalization for asthma or COPD (0.6% vs 0.6%).
- Results of the primary outcome were similar across all prespecified subgroups.

## STUDY DESCRIPTION

### Setting

- This was a pragmatic, randomized, open-label, intention-to-treat, superiority trial at 45 centers in Sweden, Estonia, and New Zealand.
- Inclusion criteria: Adult patients within 1 week of MI with obstructive CAD on angiography and EF > 50%.
- Exclusion criteria: Patients with an additional indication for or contraindication to beta-blockers were ineligible.

### Methods

- Participants were randomized in a 1:1 ratio to receive beta-blockers in the hospital and receive a prescription for cardioselective beta-blockers (metoprolol or bisoprolol) after discharge.
- Control patients were discouraged from using beta-blocker unless other indications arose.
- Data was collected from national registries. Diagnoses of myocardial infarction were verified by principal investigators using a standardized checklist.
- This study was unblinded. The investigators commented that blinding was not deemed feasible because of the pragmatic, registry-based design.
- The study was powered at 80% to detect a 25% reduction in composite primary outcome, corresponding to a 0.9% absolute risk reduction.

## STUDY QUALITY AND APPLICATION TO PATIENTS

- Strengths of the study include its large scale, randomization, and intention-to-treat design.
- Limitations included lack of blinding, majority white male patient population, moderate rates of crossover, outcomes collected via registry, and lack of central adjudication committee.
- Almost all participants received indicated medical therapy for secondary prevention.
- The findings are consistent with the ABYSS trial which reported similar results for death and MI.^4^
- This study suggests that beta-blockers are not required therapy for post-MI patients with preserved EF > 50%. Meta-analyses of contemporary RCTs support continued use in those with EF 40–49% for reducing all-cause mortality, recurrent MI, and heart failure.^4^
- Findings may not apply to patients who did not undergo PCI or to those at highest risk, including patients with three-vessel disease.
- Beta-blockers remain a first-line treatment for stable angina and heart failure with reduced ejection fraction.

## Data Availability

All data analyzed are contained within the cited manuscript.
